# Techniques Used to Determine Extreme Wave Heights from the NESS Data Set

**DOI:** 10.6028/jres.099.041

**Published:** 1994

**Authors:** Marc A. Maes, George Z. Gu

**Affiliations:** Department of Civil Engineering, The University of Calgary, Calgary, Alberta T2N 1N4; Dallas E&P Engineering (Mobil Research and Development), Dallas, TX

**Keywords:** extreme value analysis, extreme wave heights, hindcast, wave statistics

## Abstract

The objective of the present paper is to explore the application of different extreme value procedures to a selected grid point in the NESS (North European Storm Study) data set. For the purpose of the present benchmark study, each of the participants was invited to submit estimates of a significant wave height with a high return period at a given grid point in the North Sea, together with a detailed description and a rationale of the approach used in deriving extreme wave heights. Submissions from five industry groups were received, compared, and benchmarked against one another. This analysis leads to a number of conclusions regarding the actual use and misuse of extreme value techniques in the fields of engineering and oceanography.

## 1. Introduction

The NESS project [1] was funded by eleven participants and conducted by a consortium of five research institutes in Europe. The NESS users group built up considerable experience in the field of extremal value (EV) applications. (For a list of acronyms see Appendix A.) Valuable input was received from recognized experts in EV theory. It should therefore be made clear that the objective of the present comparison is not to review/criticize the use of particular extreme value techniques. Rather, the objective is (1) to reflect upon the diversity of the modelling assumptions and the procedures used to determine extreme wave heights, (2) to report on how the different groups set out to deal with difficult issues such as data reduction, statistical and parameter uncertainty, hindcast model uncertainty, and the consideration of measured data, and (3) to seek constructive guidance in this area from the extreme value specialists present at the NIST/Temple University EV Conference.

## 2. The NESS Data Set

The wave model used in the North European Storm Study (NESS) is an adaptation of the model HYPAS (Hybrid Parametrical Shallow Water wave model by Günther and Rosenthal [2]). The model results used here are from the “fine” grid model, which has a resolution of 30 km and output available every 3 h. Data are available for the periods: 1) 25 continuous 6 month (October–March) winter periods for the winters of 64/65 through 88/89; 2) three continuous 6 month (April–September) summer periods for the summers of 77 through 79; 3) continuous data for the month of April 85; and 4) 40 discrete storm periods in the April-September summer periods between 1968 and 1988. The significant wave height, *Hs*, from the model is based on the spectral definition of *Hs*, i.e., four times the square root of the spectral variance. It is assumed that *Hs* is representative of a 3 h average sea state in a 30 km by 30 km square around the grid point, and that all storms, which would have any effect on annual extreme values of *Hs*, are included in the data set.

## 3. Benchmark

The NESS grid point used in the present studies, was selected to be a location in the Forties area of the North Sea with latitude 57.777° N and longitude 0.952° E and a water depth of about 100 m. The NESS participants were invited to provide, as a minimum requirement, their “best” estimate of a 100 year return period significant wave height, *Hs-100*, for this grid point together with a short writeup describing how and why a particular EV procedure was used. Five industry groups submitted contributions; for the purpose of this paper, it was agreed not to identify the contributors; they will be referred to as groups A, B, C, D, and E. All the contributors exceeded the basic requirement of providing a 100 year return wave. Particular emphasis was given to the question of how to account for the uncertainty associated with the hindcast model itself. The contributors’ supererogation should not come as a surprise—many of the analysis procedures are coloured by subjective choices and assumptions: it is very much up to individuals to decide what looks good, what techniques are appropriate, how they should be used, and which numbers will finally be acceptable.

The emergence of the NESS database in the North Sea is not the cause of the divergence of extreme value analysis methods. To date extremal analyses have been based on available measured wave data sets —each with their own degree of accuracy and length of record. Only one attempt has been made to use the measured data in a consistent manner in estimation of extremes. The results of that pioneering effort are reported in the U.K. OTH 89 300 Supporting Document [3]. Because those results were intended to provide “indicative” values of extreme environmental criteria, implicit interpretations were made, for example in extreme value extrapolations, to reduce the risk that the results might be underestimates. It was also accepted at the time that a case for other values could be made. For reference, the results at Forties in that document provide an *Hs-100* of 14.3 m.

In the following five Sections, the five benchmark study contributions are summarized. Acronyms are used to denote the several cdfs used by the contributors; to avoid confusion caused by unclear terminology, the distributions corresponding with each acronym are listed in Appendix A. Whenever “storm peaks” are used in a subsequent analysis, the contributors resort to the same peak identification procedure: peaks are identified by determining the maximum wave *Hs* within a moving 18 h window—the average duration of a storm event.

## 4. Contribution A

Two basic techniques are used. The first one (A1) consists of fitting all 3 hourly data to a (three parameter) Weibull (W3), a Gumbel (G), or an FT3 distribution (the extreme value distribution with an upper bound) using either MOM or LS. The selection between the two distributions is made on the basis of individual judgment or a goodness-of-fit (GOF) criterion.

The second technique (A2) is a peak over threshold (POT) analysis of all peak storm event values exceeding a given level. The threshold data are fitted to either an exponential cdf (EXP) or a two parameter Frechet cdf (F2); MOM or LS are used to estimate the parameters of these conditional distributions; a plotting position *i/n* + 1 is used in the case of LS, but it is not clear in which direction errors were considered. Selection is based on best visual fit or GOF. Threshold upcrossings are assumed to be Poisson distributed, with λ estimated as the average number of storms (with peak wave exceeding the threshold) per year. The NESS results for the selected gridpoint are given in Table 1, together with results of the same analysis performed on a set of proprietory measured wave data from the same area, at Forties.

The 100 year return values of the distributions selected on the basis of a good quality fit are now averaged, and it appears that there is a substantial difference of some 2.6 m between NESS and measured data (Table 1). Three corrections are applied:

1. The measured data consist of *Hs* estimates taken at a point (“spot data”) over a 20 min sample interval and recorded at hourly or 3 hourly intervals; a “new” data base was created by converting them to 3 h averages similar to NESS. Extreme value analyses on the original set and the converted set were compared and it was found that the “3 h average” data consistently gave lower estimates of 100 year *Hs* extremes in comparison to “spot” data. The variation ranges from 0.5 m to 1.2 m, with an average value of 0.9 m, or about 6% (see Table 1).
2. The NESS database finishes at the end of March 1989. Some storms in the North Sea since that date have been very severe; indeed the most severe storm measured at Forties occurred in December 1990, when an *Hs* of 11.6 m was recorded. The effect of this missing data in the NESS archive was assessed, albeit indirectly, by examining the effect that the equivalent period has on extrapolations of the Forties measured data. From the various analyses performed for the above periods, the effect on 100 year *Hs* estimates of including data recorded in the period April 1989 to May 1992 ranged from increases of 0.2 m to 0.9 m. The average increase across the analyses was 0.6 m, or about 5% of the shorter period estimate.
3. Both regression and extreme value analyses have been performed on selected overlaps between NESS and measured data. The regression analyses revealed that the mean NESS wave height was some 10% higher than the measured wave height, but when extrapolated to extremes, the 100 year *Hs* estimates from NESS were between 0.5 m and 2.0 m lower than extrapolations from the measured database, with an average difference of 1.2 m. This apparent offset could be due to a wide range of factors, many (if not all) of which are under investigation by the NESS User Group at the time of writing.

All three corrections are captured in Table 1. The conservative view taken in applying the three corrections is indicative of the safety margin associated with the final estimate *hs*-100 = 12.6 m; however, no specific uncertainty band is provided.

## 5. Contribution B

Group B’s procedure for wave criteria determination for Forties is as Follows:

1. Extract storm peak data at the reference grid-point with a threshold of *Hs* = 6.0 m and an 18 h window (298 storm peaks).
2. Select the Annual Extreme Value (AEV) for each of the 25 years from the 298 peaks. The 25 values range from 6.9 m to 9.9 m. The reasons for using AEV instead of POT method are described to be the following:
  - AEV shows consistently better fits than POT (higher correlation coefficients, smaller mean square errors in the case of LS, and larger likelihood functions in the case of MLE);
  - The extremes from AEV are not influenced by the threshold, i.e., they are less subjective;
  - Extremes based on AEV method tend to be higher than POT (more conservative);
  - In the North Sea, due to the high frequency of storms, the highest *Hs* in a year does represent the wave severity for the year in most cases, whereas for POT, when calculating extremes for various thresholds, it is sometimes found that the storm frequency for the best fit is less than 1.0/year, less than that for AEV.
3. The 25 Annual Extreme Values are fitted to six distributions: G, BM, FR3, FT3, W3, EXP, using two estimation methods: LLS and MLE. All six LLS fits are very good since the correlation coefficients all exceed 0.98. Only three MLE fits are considered acceptable: this was judged on the basis of the relative magnitudes of the likelihood function. The range of the 100 year *Hs* given by the nine good fit cases (6 LLS and 3 MLE) is from 10.6 m to 11.5 m (Table 2). Since Gumbel is theoretically sound for annual extremes and the Gumbel LLS gives an excellent fit, it was decided that Gumbel LLS would be used throughout the analysis. Plotting positions for LLS are *i/n +* 1 and squared errors On *Hs* are minimized.
4. To take consideration of possible bias in NESS, measured, smoothed storm peaks at the Forties location are plotted against the corresponding peak *Hs* from NESS. Only peaks exceeding 6.0 m are considered. A regression analysis yields the best fit linear function
  Hs,measured=0.63+0.9746Hs,NESS (1)
  with a standard deviation of 0.93 m.
5. The 298 NESS peaks are adjusted on the basis of Eq. (1), and the analysis steps 2 and 3 are repeated; this is shown in the second column of *Hs-100* values in Table 2.
6. Scatter is now considered by adding random errors to the adjusted 298 storm peaks. Random errors are generated using a Monte Carlo simulation assuming a normal distribution with a standard deviation of 1.0 m (1.0 m is selected as a round-off value of the regression model error of 0.93 discussed above). An AEV LLS Gumbel analysis is performed on the *Hs* data containing random errors. After 10,000 simulations the average *Hs-100* is 12.4 m with a sample standard deviation of 0.66 m.
7. The proposed 100 year return period *Hs* is taken to be 12.4 m.

## 6. Contribution C

Group C established the following EV procedure for NESS data. The method accounts for spatial spreading using neighbouring grid points, but this aspect of the procedure will not be described. To start with, the 2 parameter Weibull cdf (W2) is fitted to the cumulative frequency distribution of all the data. In practice, however, a best fit is sought for the top 10% of these data. MML is used to estimate the parameters. POT is suggested as an alternative method for a finer 10 × 10 km grid, but not for the 30 × 30 km grid under consideration.

The NESS extremes are now corrected to take into account hindcast model uncertainty by applying all of the following techniques:

C1: add random Gaussian noise at 5%, 8%, and 10% to the W2 cdf (the KESPL method) and record the increase of *Hs-100*.
C2: obtain short return period quantiles, specifically those having exceedance probabilities equal to 12*/k* and 1*/k*, where *k* = 365 × 8/2 is approximately the number of NESS data per year; then multiply these two values with 1.86 and 1.40, respectively, (the RATIO method). The idea of scaling short return period values to 100 year estimates using factors obtained from measurements, originates from the so-called Jenkinson method used by the UK Met Office for deriving extreme wind speeds.
C3: use a linear equation to transform both W2 parameters to “equivalent measured” parameters (the PARAMETER method). The equation derives from an existing regression between hindcast and measured data.

The final step is to interpret the results obtained and to compare them with all available measured data (Table 3). In the case of Forties, the *Hs-100* based on measurements was found to be 13.29 m; this indicates that a 8% noise level is appropriate under C1, and a 1 year ratio method under C2. The 100 year return values for C2 and C3 are averaged, and this value is then averaged with C1. This is considered to be the best “equivalent measure” *Hs-100*. Finally, this result is averaged once more with the direct NESS estimate and a correction factor of 1.03 is applied to take into account that NESS covers only the 6 month winter period in each year. The results in Table 3 should be used with caution: certain values are valid for the averages of 5 gridpoints (including our reference point) covering the Forties area.

## 7. Contribution D

Group D’s Method is a POT method of storm peak values. The threshold is varied in increments of 0.1 m, until a good visual fit is obtained to the following distributions: Gumbel (G), Exponential (EXP), two-parameter Weibull (W2), Pareto (P), lognormal (LN), and generalized gamma (GG); parameters are estimated using LS or MML, except in the last case where a MOM based on Stacy and Mirham [4] is used. The empirical cdf is taken to be *i/n* * + 1, where n* is the number of data exceeding the threshold.

In this particular case, little variation in *Hs-100* was detected when the threshold was varied from 7.0 m to about 7.9 m, and reasonable fits were obtained using G, GG, and LN. The final selection of a threshold of 7.8 m was guided by the principle that a POT analysis should ideally be conducted using (approximately) the top 40 data. The best visual fit on a Gumbel plot is obtained by the GG (Table 4). The estimate of *Hs-100* is 10.7 m, which is rounded to 11.0 m.

Measured storm peak data at the Forties are taken into account by multiplying the NESS estimate by 1.07. The value of this multiplication factor is justified on the basis of the following two considerations:

- perform a peak-to-peak scatter plot (measured vs NESS): the best fit regression line forced through the origin has a slope equal to 1.07;
- a POT analysis (threshold = 7.0 m) is conducted on the measured data and on the corresponding NESS data (i.e., the NESS data occurring simultaneously with the measured data). The 100 year return period on the former turns out to be 7% greater than the NESS *Hs-100;* the generalized gamma cdf was also used for this purpose.

Table 4 summarizes the intermediate values and shows Group D’s best estimate *Hs-100* = 12.0 m.

## 8. Contribution E

POT storm are fitted to one of three distributions that are left-truncated below the threshold *x*_0_: Gumbel truncated (GT), Frechet truncated (FR2T), and Weibull truncated (WT). In the present application, however, the second cdf failed to give a good fit and was discarded. The likelihood expressions involve three parameters: the two basic parameters, together with *x*_0_. In practice, the MML is applied to determine the two basic parameters, given *x*_0_. The associated 95% confidence bands on the corresponding 100 year return values are determined using the 2×2 observed information matrix, given *x*_0_. The candidate distribution is selected on the basis of (1) the correlation coefficient for LS residuals in the *Hs* direction (usually > 99%), (2) the mean error on cumulative probabilities (generally ≤ 0.05), (3) the mean square error on cumulative probabilities (generally ≤ 5 0.01), and (4) visual assessment.

To determine the threshold *x*_0_, the above procedure is repeated in order to find a range of thresholds over which both the goodness-of-fit statistics, as well as the extrapolated design value are stationary. The selection process is guided by the condition that the annual storm frequency at the site should be between 1.0 (0.5) and 3.0 (4.0). This frequency is proportional to the inverse of the number of peaks exceeding *x*_0_. The half range of the two parameter confidence interval obtained using the MML procedure for the selected *x*_0_, is now added to the *Hs-100* value. The addition of the half-range confidence interval derives from the concern noted in Contribution A that the 3 h average hindcast data overly smooths storm peaks as compared to the 20 min “spot” measured data. In the comparisons to measured data at three North Sea sites which have been performed to date, use of the confidence interval results in an unbiased extrapolation at both the 40 year and the 100 year return period.

As with the previously discussed procedures, the impact of several severe storms which have occurred after the NESS hindcast period was also considered. Measured data from those storms were used to artificially extend the hindcast database. A repeat of the above procedure using the extended database resulted in an increase in the extrapolated 100 year wave height by 0.7 m. The best estimate of *Hs*, shown in Table 5, including the confidence interval and consideration of post-Ness storms, is 12.0 m.

## 9. Summary

Five NESS participants were asked to provide their best estimate of the 100 year return significant wave height at a given grid point in the North Sea. We cannot help being pleasantly surprised with the astonishing array of approaches used by the participants: all submissions attest to the fact that the contributors have an expert understanding of the NESS statistics and the extreme value methods needed to formulate engineering design criteria. Our second impression is equally compelling: not withstanding the diversity of selected EV methods and the variety of subsequently applied “adjustments/corrections,” it is interesting to observe that the recommended *Hs-100* values lie very close to one another.

Table 6 summarizes the final results. The first row lists the *Hs-100* obtained from a direct EV analysis of the NESS data: all values submitted can essentially be rounded off to the same number: 11.0 m. This value may be contrasted with the aforementioned reference approach (R), which was seen to result in a significant wave height of 14.3 m.

## 10. Evaluation

Each Submission contains a fair number of steps that require the use of good judgement and subjective reasoning. Several issues are simply not amenable to quantitative evaluation. For instance, the reason for selecting a particular approach may be that it is a given group’s standard way of dealing with extreme value problems, or it may be an approach strongly favored by one or more people, or it may be a series of procedures developed over the years, which enjoys a history of frequent and successful use. At the same time, each group must attempt to derive a result that is theoretically defensible as well as one that will in all likelihood be acceptable to the outside world (management, designers, regulatory agencies, etc.)

Consequently, there are several aspects of the submissions that are difficult to interpret. Keeping these limitations in mind, it seems reasonable to identify the following basic criteria to assess the quality of a particular approach:

1. How practical and clear is the suggested approach? A convincing procedure must be logical and simple to use.
2. Is the method theoretically sound and does it lead to accurate results? Is it based on recognized statistical techniques and proven results from extreme value theory?
3. Can the method be generalized easily to other gridpoints and locations or is it very dependent on a particular data structure? How wide is its range of applicability?
4. How sensitive is the method to assumptions regarding data, distribution types? Is the method robust? Can confidence intervals easily be constructed? Is parameter/statistical uncertainty taken into account?
5. Does the method allow for adjustment based on measured data; is the hindcast model uncertainty taken into account?
  In fact, two questions should be considered:
  (5a) When measured data are available at the site, can a suitable procedure be used to incorporate them in any way? and;
  (5b) When no measured data are available at the site, can the intrinsic hindcast model uncertainty be accounted for in a NESS EV analysis?

A detailed evaluation is not attempted, but it is felt that most of the methods used would get a fine score against each of the above criteria, with the exception of criterion (5b). This is due to the lack of a consistent technique to account for the intrinsic hindcast model uncertainty, even in the absence of measured data. Another weakness would be reflected in criterion (4): parameter uncertainty and/or short data uncertainty should be addressed in extrapolating to high return values.

On a theoretical level, we feel somewhat uneasy about the use of “cumulative” data (as opposed to working with storm peaks): the implication on estimating high extreme values is not clear. As far as distribution choices are concerned, three considerations jump to mind. First, we are somewhat surprised that no contribution included the (3 parameter) generalized extreme value cdf in the analysis; this is a particularly flexible distribution and it could virtually be used on its own to model a wide range of tail behaviors. By the same token, no attempt was made to look at an analysis based on seasonal extremes, month-by-month extremes, and the effective use of more than just one high order statistic (for instance through the use of the i dimensional generalized extreme value cdf). Weighted least squares also failed to be selected as a convenient way to correct tail behavior. With regard to the POT analyses, it is somewhat puzzling to see that distributions which would not be expected to yield good fits were included in the analysis (one would expect POT density functions to be monotonically decreasing starting at the threshold).

Further discussion is needed to investigate the quality of the different approaches.

## 11. Problem Issues

In the course of evaluating the different submissions, it becomes clear that there are a number of grey areas with issues that will need to be dealt with at some point in the future. Guidance needs to be sought from experts in EV analysis and from experienced oceanographers and engineers with regard to these matters. The following list may not be complete, but it does contain a set of both general and particular issues identified in the process of analyzing the five contributions:

### 11.1 Issues related to EV analysis

1 Selection of storm peaks from 3 hourly data; smoothing/interpolation of peaks; storm duration: can the 18 h window criterion be relaxed?
2 Least Squares Methods: plotting position to be used, particulary in the case of upper tail analysis; in which direction should errors be considered: variable, log (exceedance probability), weights?
3 POT: How many data are needed; How does the threshold need to be selected (almost all of the contributors used different criteria); In deriving EVs, is it preferable to use quantiles simply on the basis of an adjusted exceedance probability or on the basis of a compound Poisson cdf?
4 Develop means to construct confidence intervals associated with some of the more complicated methods. Only contributor E made an attempt to account for parameter uncertainty.
5 When using the “cumulative” (all data) approach, assess the impact of correlation between peaks, particularly when only a small percentage of the top data is used.
6 Evaluate the impact of discontinuous data on determining *r* year return periods; in the particular case of AEV, what is the impact of using 6 month (winter) extremes?

### 11.2 Additional Issues

7 Measured Data; the various used/proposed methods require a detailed examination. Clarification and consensus is needed on how to “combine” hindcast and measured data. Some of the approaches reflect a sense of “we know what number we want to get close to, so let’s select a method that will get us there”; this arbitrary aspect should be addressed.
8 Spatial Spreading; this issue was not part of the present analysis, but contributor C showed that any method should also be applicable to a series of gridpoints, rather than just one gridpoint.
9 Inclusion of recent storms and/or recently observed high *Hs* values in a NESS extreme value analysis. Only contributor A explicitly addressed this seemingly important issue.

## Figures and Tables

**Table 1:** Group A results; 100 year return period *Hs* (m)

A1	NESS (3 hourly data)	Measurements (“spot” data)
W3, LS	11.4^a^	13.0*
G, LS	13.2	13.6
FT3, LS	10.3^a^	12.3
W3, MOM	11.9	12.8*
G, MOM	16.3	14.0

A2	50 storm peaks with *Hs* >7.6 m	52 storm peaks with *Hs* > 7.6 m

FR2, LS	10.7	14.2
EXP, LS	10.9^a^	14.1^a^
F2, MOM	10.4	13.3
EXP, MOM	10.5	13.5^a^

Average of *Hs* with good quality fit	10.8	13.4

Convert “spot” to “3 hourly”	+ 0.6	− 0.9
Account for 1989–1992 data	+ 1.2	
Offset NESS/measurements	12.6	12.5
Adjusted estimate		

Final estimate	12.6	

a Good quality fit.

**Table 2:** Group B results; 100 year return period *Hs* (m)

	NESS (3 hourly data)	NESS with Linear adjustment
G, LS	11.0	11.4
BM, LS	10.7	11.2
FT2, LS	11.1	11.4
FT3, LS	11.0	11.3
W3, LS	10.8	10.9
F, MLE	10.6	11.1
FT2, MLE	10.8	11.3
W3, MLE	10.6	10.9
Incl. Random Errors (STD = 1.0 m)		12.4

Final estimate	12.4	

**Table 3:** Group C results; 100 year return period *Hs* (m)

1. Directly from NESS (W2, MLE)	11.3
2. Measured data available	13.3^a^
3. C1 using noise at 8%	11.4^a^
4. C2 using 1:100 ratio	12.4
5. C3	12.4
6. Best “Equivalent Measured”: 0.5 C1 + 0.25 C2 + 0.25 C3	12.2^a^
7. Final estimate = 1.03 × average of 1 & 6	12.2^a^

a These values are averages of 5 grid points located in Forties.

**Table 4:** Group D results; 100 year return period *Hs* (m)

POT with threshold of 7.8 m (n* = 37)	
1. Gumbel using MML	9.8
2. Gumbel using Ls	10.4
3. LN using MML	9.7
4. LN using LS	9.9
5. P using MML	13.0
6. GG using MOM	10.7^a^
7. NESS best estimate (round off of 6.)	11.0
8. Correction based on Measured Data: (7.)^a^ 1.07	12.0

a Indicates best fit.

**Table 5:** Group E Results; 100 year return period *Hs* (m)

Threshold × 0 (m)	Number of points > × 0	Annual storm frequency	*Hs-100* (m)	
			Fit to GT	Fit to WT
Stationary range				
7.00	118	4.7	10.8	10.6
7.15	92	3.7	10.6	10.4
7.25	81	3.2	10.7	10.5
7.35	68	2.7	10.6	10.4
7.45	56	2.2	10.5	10.3
7.55	50	2.0	10.6	10.4
7.65	44	1.8	10.6	10.5
7.75	37	1.5	10.6	10.6
7.85	29	1.2	10.7	10.3

Average			10.6	10.4

Best NESS estimate			10.5	

Add 95% C1 as described			11.3	

Add 0.7 m to account for post NESS storms			12.0	

Final design value			12.0	

**Table 6:** Summary of Recommended *Hs-100* (m)

	A	B	C	D	E	R
Value based exclusively on NESS data	10.8	11.0	11.3*	11.0	10.5	14.3
Recommended value including all corrections/uncertainties	12.6	12.4	12.2*	12.0	12.0	

## 12. Appendix A. Acronyms Used for Distribution Functions and Analysis Methods

A,B,C,D,E: the 5 contributing groups
AEV: Annual Extreme Value (Method)
BM: Borgman cdf
  $$\begin{array}{cc} F(x)=\exp\left[-\exp\left(-\frac{x^{2}-a}{b}\right)\right] & x>0;\;b>0 \end{array}$$
EXP: exponential cdf:
  $$\begin{array}{cc} F(x)=1-\exp\left(-\frac{x-a}{b}\right) & x>a;\;b>0 \end{array}$$
EV: Extreme Value
FR2: 2 parameter Frechet (or Fisher-Tippett Type 2, or log extremal) cdf:
  $$\begin{array}{cc} F(x)=\exp\left[-{\left(\frac{x}{b}\right)}^{-c}\right] & x>0;\;b,\;c>0 \end{array}$$
FR3: 3 parameter Frechet (or Fisher-Tippett Type 2, or log extremal) cdf:
  $$\begin{array}{cc} F(x)=\exp\left[-{\left(\frac{x-a}{b}\right)}^{-c}\right] & x>0;\;b,\;c>0 \end{array}$$
FR2T: Left-truncated Frechet cdf
FT3: the Fisher-Tippett Type 3 (or, the inverted Weibull) cdf:
  $$\begin{array}{cc} F(x)=\exp\left[-{\left(\frac{a-x}{b}\right)}^{c}\right] & x<a;\;b,\;c>0 \end{array}$$
G: Gumbel cdf
  $$\begin{array}{c} F(x)=\exp\left[-\exp\left(-\frac{x-a}{b}\right)\right]\\ \;-\infty <x<+\;\infty ;\;b>0 \end{array}$$
GG: 3 parameter generalized gamma cdf with probability density function:
  $$\begin{array}{c} f(x)=\frac{\lambda \beta }{\Gamma (\alpha )}\;{\left(\lambda x\right)}^{\alpha \beta -1}\exp\;[-{\left(\lambda x\right)}^{\beta }]\\ x>0;\;\alpha ,\;\beta ,\;\lambda >0 \end{array}$$
GOF: goodness-of-fit
GT: Left-truncated Gumbel cdf
HYPAS: Hybrid Parametrical Shallow Water Wave Model
MOM: method of moments
MML: methods of maximum likelihood
MLE: maximum likelihood estimate
LN: 2 parameter log-normal cdf (Log of the variable has a normal cdf)
LS, LLS: least squares, linear least squares
NESS: North European Storm Study
P: Pareto cdf
  $$\begin{array}{cc} F(x)=1-x^{-\beta }, & x>1;\;\beta >0 \end{array}$$
POT: peak over threshold
R: the reference approach in the Guidance Notes
W2: the 2 parameter Weibull cdf (or the FT3 for minima)
  $$\begin{array}{cc} F(x)=1-\exp\left[-{\left(\frac{x}{b}\right)}^{c}\right] & x>0;\;b,\;c>0 \end{array}$$
W3: the 3 parameter Weibull cdf (or the FT3 for minima)
  $$\begin{array}{cc} F(x)=1-\exp\left[-{\left(\frac{a-x}{b}\right)}^{c}\right] & x>a;\;b,\;c>0 \end{array}$$
W2T: Left-truncated Weibull cdf
